# Accumulation of Amino Acids and Flavonoids in Young Tea Shoots Is Highly Correlated With Carbon and Nitrogen Metabolism in Roots and Mature Leaves

**DOI:** 10.3389/fpls.2021.756433

**Published:** 2021-11-18

**Authors:** Jianwei Liu, Meiya Liu, Hanhan Fang, Qunfeng Zhang, Jianyun Ruan

**Affiliations:** ^1^Key Laboratory for Plant Biology and Resource Application of Tea, The Ministry of Agriculture, Tea Research Institute, Chinese Academy of Agricultural Sciences, Hangzhou, China; ^2^Agricultural Technology Extension Center of Fuyang, Hangzhou, China; ^3^Fuyang College, Hangzhou, China

**Keywords:** nitrogen, metabolomics, amino acid, tea root, flavonoid, tea quality

## Abstract

The quality of tea product and the metabolism of quality-related compounds in young shoots are significantly affected by the nitrogen(N) supply. However, little is known of the metabolic changes that take place in tea roots and mature leaves under different supplies, which has a large effect on the accumulation of quality-related compounds in young shoots. In this study, young shoots, mature leaves, and roots under different N conditions were subjected to metabolite profiling using gas chromatography and ultraperformance liquid chromatography, coupled with quadrupole time-of-flight mass spectrometry. The contents of free amino acids (e.g., theanine and glutamate) involved in N metabolism were significantly greater under high N than under low N, while a high N supply reduced soluble sugars (e.g., glucose) in all three tissues. Organic acids (e.g., malate, fumarate, α-ketoglutatare, and succinate) involved in tricarboxylic acid cycle remarkably increased as the nitrogen supply increased, which confirms that carbon (C) allocation was restricted by increasing the nitrogen supply, especially in mature leaves. RT-PCR results indicated that gene expression related to nitrogen assimilation significantly increased in roots with increasing nitrogen supply, which had a significant positive relationship with the level of free amino acids in young shoots. In addition, the expression of most genes involved in flavonoid synthesis was significantly upregulated under conditions of low nitrogen supply relative to high nitrogen supply in young shoot and roots. These data suggest that enhanced assimilation of N in tea roots and the coordinated regulation of C (sugars, organic acids, and flavonoids) and N(amino acids) in mature leaves can lead to a high accumulation of amino acids in young shoots. Furthermore, as the N supply increased, more C was partitioned into compounds containing N in mature leaves and roots, resulting in a decrease in flavonoids in young shoots. In conclusion, the accumulation of amino acids and flavonoids in young tea shoots is highly correlated with carbon and nitrogen metabolism in roots and mature leaves.

## Introduction

The growth and quality of tea are strongly influenced by the nitrogen supply, which influences metabolites, enzymes, and genes (Bryan, 2008). The flavor of tea is largely affected by the abundance of chemical constituents, including polyphenols, caffeine, amino acids, and vitamins, and their relative composition in young shoots. For example, quality green tea is characterized by high contents of free amino acids with appropriate concentrations of catechins and caffeine. The chemical compositions of tea are affected by many factors, such as the environment (soil, altitude, nutrient supply, and temperature) and the genetic background (Liu et al., 2016). Ruan et al. (2010) found that concentrations of free amino acids increased with increasing N supply in tea plants, and the makeup of free amino acids shifted toward those characterized by low C/N ratios (arginine, glutamine) and away from theanine, a unique non-protein free amino acid. A high N supply significantly reduces the concentrations of polyphenols and soluble sugars in young shoots, and more C skeletons are used for free amino acids under such conditions (Ruan and Sattelmacher, 2007; Ruan et al., 2010). Li et al. (2017) investigated transcriptomes in two tea varieties using RNA-seq and the contents of amino acid under different N treatments. They identified 196 and 29 differentially expressed genes in roots and leaves, respectively. *AMT*, *NRT*, and *AQP* were the key genes for N uptake, and *GOGAT* and *GS* were key for N assimilation. Under conditions of N deficiency, the amounts of organic acids involved in the tricarboxylic acid (TCA) cycle decrease, together with metabolites (e.g., amino acids, soluble sugars, and sugar alcohols); likewise, proteins and genes are profoundly altered (Amiour et al., 2012). Some differences appear between roots and shoots in terms of metabolic adaptation to response to N starvation (Krapp and Daniel, 2011). Catechin biosynthesis-related genes (*PAL*, *CHS*, *CHI*, and especially *DFR*) exhibit higher expression levels under nitrogen-free conditions (Kai et al., 2015). Fan et al. (2019) described the mechanism of the re-allocation of N and carbohydrates from source leaf to flower in tea plants using metabolomics and genes involved in autophagy, protease, H + /sucrose symporters, amino acid permease, glutamine synthetase, and asparagine synthetase. Huang et al. (2018) found that nitrogen-deficient tea plants accumulate diverse flavonoids, while N-supplied tea plants significantly increase their proline, glutamine, and theanine levels in young shoots. Zhang et al. (2017) reported the molecular mechanisms related to free amino acid metabolism using transcriptome and metabolomic analyses of the Huangjinya tea cultivar. Linking gene expression to metabolite synthesis and accumulation is a significant challenge for improving tea quality. In this study, we hypothesized the accumulation of quality-related compounds in young tea shoots was highly affected by the metabolism and the expression of key genes in mature tea leaves and roots, especially under different N supply conditions. We hope that this work will provide a framework for an improved understanding of the molecular and metabolite mechanisms of the accumulation of quality-related compounds in young shoots affected by N supply, as well as metabolism in roots and mature leaves.

## Materials and Methods

### Plant Cultivation

Rooted cuttings (cv. Longjing43) aged 1 year that were pre-cultivated in diluted nutrient solution (1/8 full strength) for 1 year were transferred to a pot with 4 L full-strength nutrient solution, containing the macronutrients (mmol/L) (P 0.1, K 1.0, Ca 0.8, and Mg 0.4) and the micronutrients (μmol/L) (EDTA-Fe 6.3, Mn 1.5, Zn 1.0, Cu 0.2, B 10, and Mo 0.5). The pH of nutrient solutions was continuously titrated to 5.0 with H_2_SO4 and NaOH, using a custom-built pH stat system with an accuracy of about ± 0.2 pH (Ruan et al., 2010). The cationic nutrients were supplied as sulfate salts, and chloride (0.05 mmol/L) was supplied in the form of CaCl_2_. All pots were evenly placed in the climate chamber. The air temperature and relative humidity were maintained at 26/22°C in the photo/dark period and 70%, respectively. The photo/dark period was 14/10 h, and the light intensity was 200 mmol m^–2^ s^–1^. Rooted cuttings (*n* = 192) were divided into three treatment groups, with eight pots in each group, and eight tea seedlings per pot. The three treatments contained different concentrations of N at 0.3 (N1), 0.75 (N2), or 4.5 (N4) mmol/L, provided as NH_4_^+^ and NO_3_ (NH_4_^+^:NO_3_ of 3:1). Nutrient solutions were replaced each week (Ruan et al., 2010). After 4 weeks, the roots, mature leaves, and new shoots of the tea plants were collected (each sample is a mixture of 16 plants) and frozen in liquid nitrogen (Liu et al., 2016). The samples of young shoots consisted of two expanding leaves and a bud.

### Quantitative Real-Time PCR Analyses and Metabolomic Analyses Based on UPLC-Q-TOF/MS and GC × GC-TOF/MS

Samples of young leaves and roots have been used for gene expression analysis. Total RNA was extracted using an RNA Plant Plus kit (Tiangen, China). Quantitative real-time polymerase chain reaction (RT-qPCR) was performed on an Applied Biosystems 7300 machine (Carlsbad, CA, United States). For each target gene, reactions were performed in triplicate. Relative transcript levels were calculated against those of the internal control *GAPDH* using the formula 2^–Δ^ ^*Ct*^. The procedures for qPCR analyses as described by Zhang et al. (2017) were followed.

The freeze-dried samples were subjected to metabolomic analyses using an ultra-performance liquid chromatography device, coupled with a hybrid quadrupole orthogonal time of flight mass spectrometer (UPLC-Q-TOF/MS) and a two-dimensional gas chromatography device coupled with a time a flight mass spectrometer (GC × GC-TOF/MS, Agilent 6890N, Agilent Technologies, Santa Clara, CA, United States, Pegasus HT, Leco Co., St. Joseph, MI, United States). The procedures followed Liu et al. (2016). For metabolomic analysis based on UPLC-Q-TOF/MS, the metabolites in plant samples were extracted with 1 mL of a solvent mixture of 75% methanol and 1% formic acid for 10 min in an ultrasonic bath and then centrifuged at 12,000 rpm for 10 min. After filtration through a 0.22-μm PTFE filter, a 2 μL extract was injected into an HSS T3 column on a UPLC-QTOF/MS (ACQUITY UPLC/Xevo G2-S Q-TOF/MS, Waters Corp., Milford, MA, United States). For metabolomic analysis based on GC × GC-TOF/MS, plant samples (100 mg) were extracted with 1000 μL of methanol/chloroform (3:1, v/v) solvent. A volume of 10 μL L-2-chlorophenylalanine (0.3 mg/mL in water) was added as internal standard. We run a mixed sample as control every 8–10 tea samples in sequence.

### Data Processing and Multivariate Data Analyses

The processing of data files from UPLC-Q-TOF/MS, including data collection, alignment, and normalization of tea metabolites, was conducted using Transomics software (Waters Corporation, CA, United States). The metabolite peaks were assigned to accurate mass measurements using online metabolite databases as described in Liu et al. (2016), and the retention times were compared to those found in the published literature (Liu et al., 2016). The data files from GC × GC-TOF/MS were processed using LECO Chroma TOF software at an S/N threshold of 500. Metabolite identification from these selected variables was achieved using NIST 05 standard mass spectral databases (NIST, Gaithersburg, MD, United States). The resulting data, containing sample information, peak retention time, and peak intensities, were normalized to the area of the IS (the IS peaks were removed afterward) and then mean-centered (Liu et al., 2016).

The pre-processed datasets of UPLC-Q-TOF/MS and GC × GC-TOF/MS were exported to SIMCA-P13.0 software (Umetrics, MKS Instruments Inc., Sweden) for multivariate analyses. The data were Pareto scaled and visualized by plotting the principal components scores in which each coordinate represents an individual biological sample. For the classification and discrimination between treatments, principal components analyses (PCAs) were carried out. Then supervised orthogonal projection to latent-structure discriminant analyses (OPLS-DA) were used to extract the maximum information from the dataset and to isolate the metabolites responsible for the differences between each group. After the multivariate approaches, the significance of each metabolite in group discrimination was measured via one-way ANOVA with Tukey’s posttest using SPSS. Potential biomarkers for grouping were selected to meet VIP > 1 and a significance threshold of *P* < 0.05 (Zhang et al., 2017). Heat maps and bar graphs were generated using the R program and Origin 8.5 (Liu et al., 2016). The Pearson correlation coefficient was used to analyze the correlations among metabolites as well as correlations between metabolites and genes, and a correlation network was drawn using Cytoscape 2.82.^1^

### Measurement of Total N, Total Carbon, and Free Amino Acids

The total N and C in the young shoots and mature leaves were determined using an elemental analyzer (Vario Max CN Analyzer, Elementar Analysensysteme GmbH, Germany) by measuring CO_2_ and N_2_ released after combustion at 950°C. An aliquot of 100 mg for each dried sample was used, and glutamic acid has been used as a standard compound. Free amino acids in young shoots samples (100 mg) were extracted by 5 mL boiling water for 5 min in 100°C water bath. Amino acid contents in the extract were measured using an automatic amino acid analyzer (Sykam S-433D, Germany). Standards were prepared from authentic reagents (Sigma-Aldrich Co., St. Louis, MO, United States).

## Results

### Influence of N Supply on Tea Plant Growth and C and N Concentrations

The level of N supply had far-reaching consequences for the growth and mineral nutrition of the tea plants. The biomass production of the roots was significantly increased by increasing N concentration in the nutrient solution (Table 1), while the leaves showed the highest biomass in the N2 treatment. The total N concentration increased, and the C/N ratio continuously decreased with increasing N supply in all tissues measured, while total C concentration did not increase significantly (*p* > 0.05).

**TABLE 1:** Biomass production and elemental concentrations and ratios in tea plants as affected by different concentrations of N in the nutrient solution.

	**N supply (mmol l^–1^)**		
	**0.3 (N1)**	**1.5 (N2)**	**4.5 (N3)**
**Biomass of (g DW plant^–1^)**			
Leaves	3.15 ± 0.07c	5.24 ± 0.18b	4.59 ± 0.06a
Root	2.24 ± 0.01c	3.54 ± 0.03b	4.09 ± 0.02a
**Elemental concentration Young shoot**			
N (%)	3.99 ± 0.06c	4.59 ± 0.12b	5.41 ± 0.10a
C (%)	40.4 ± 0.25	41.33 ± 0.25	42.95 ± 1.00
C/N	10.12 ± 0.14a	8.99 ± 0.20b	7.94 ± 0.04c
**Mature leaves**			
N (%)	2.93 ± 0.02c	4.34 ± 0.05b	4.52 ± 0.03a
C (%)	43.81 ± 0.34	43.96 ± 0.54	43.71 ± 0.27
C/N	14.95 ± 0.12a	10.14 ± 00.10b	9.66 ± 0.04c
**Root**			
N (%)	1.30 ± 0.09c	2.35 ± 0.15b	3.09 ± 0.27a
C (%)	46.59 ± 0.31	47.43 ± 0.14	47.77 ± 0.26
C/N	36.17 ± 2.81a	20.39 ± 1.31b	15.77 ± 1.09c

*Data are means ± standard error (*n* = 4). Different letters following data within a line indicate a significant (*P* < 0.05) difference in LSD tests.*

### Metabolomic Analyses of Young Shoots, Mature Leaves, and Roots

In all, 4,546 metabolites were extracted from the data sets of UPLC-QTOF/MS and GC × GC-TOF/MS. The PCA plots based on these two analytical platforms both showed a clear separation among the young shoot samples from the three periods (N1, N2, and N3) in relation to the first (explaining 46.8 and 41.2% of the variation for UPLC-Q-TOF/MS and GC × GC-TOF/MS, respectively) and the second component (explaining 13.8% and 19.9% of the variation, respectively) (Supplementary Figures 1A,B). The PCA plots based on these two analytical platforms also showed a clear separation among the mature leaves (Supplementary Figures 1C,D) and root (Supplementary Figures 1E,F). This shows that the mathematical model is successful, and the primary and secondary metabolites of young shoots, mature leaves, and roots have significant differences. These different metabolites were mainly amino acids, organic acids, carbohydrates, and flavonoids (Supplementary Table 1).

To find metabolic differences among young shoots, mature leaves, and roots under different nitrogen supplies, we chose differentially expressed metabolites according to their fold change (VIP > 1). We obtained different metabolites after screening (Supplementary Table 1).

In the primary metabolism, the contents of free amino acids related to nitrogen metabolism were significantly greater for N3 than N1. For example, the levels of theanine, glutamine, glutamate, and aspartate were higher for N3 than for N1 in young shoots (Supplementary Table 1). However, the levels of theanine, glutamate, and aspartate were higher for N3 than in N1 in mature leaves, and those of theanine, glutamine, and glutamate asparagine were higher for N3 than for N1 in roots.

The levels of soluble sugars (glucose, xylopyranose, rhamnose, lyxose, and erythritol) were lower for N3 than for N1 in young shoots, but those of others (e.g., arabitol, erythrose, galactose, and arabinose) were higher for N3, which may indicate a complex change in carbon metabolism. The levels of most carbohydrates related to carbon metabolism (e.g., erythrose, glucose, galactopyranoside, galactose, xylopyranose, glycoside, fucopyranose, and mannose) were lower for N3 than for N1 in mature leaves. Furthermore, the contents of some soluble sugars (e.g., glucose, psicose, erythrofuranose, rhamnose, fucose, and sorbose) were lower for N3 than N1 in roots (Supplementary Table 1). These results indicate that a high N supply reduces soluble sugars over the whole plant.

Complex changes were seen among the contents of different kinds of organic acids as the nitrogen supply increased. For example, the levels of succinate, ketoglutarate, butanoic acid, oxalic acid, fumaric acid, glyoxylate, and malic acid were higher for N3 than for N1, but those of citrate, isocitric, and tartaric acid were lower for N3 than for N1 in young shoots. Succinate and cinnamic acid had higher values for N3 than for N1, and but glyoxylate and tartaric acid had lower ones for N3 than for N1 in mature leaves. However, the levels of anedioic acid, cinnamic acid, and citrate were lower for N3 than for N1 in roots, whereas those of hydroxypyruvic acid, tartaric acid, oxalic acid, and fumaric acid were higher for N3 than for N1 (Supplementary Table 1).

### Influence of N Supply on Gene Expression Associated With N and C Metabolism

**FIGURE 1:**
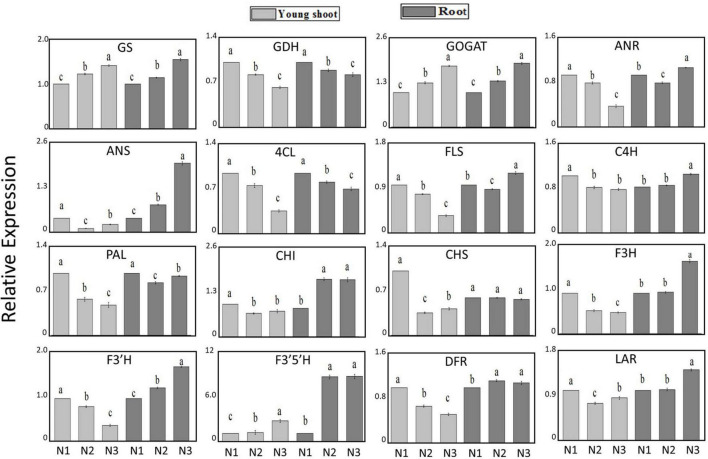
Relative expression of genes *GS, GDH, GOGAT, ANR, ANS, 4CL, FLS, C4H, PAL, CHI, CHS, F3H, F3‘H, F3‘5‘H, DFR*, and *LAR* under conditions of low (0.3 mmol/L, N1), intermediate (1.5 mmol/L, N2), and high levels of nitrogen (4.5 mmol/L, N3) in young shoots, mature leaves, and roots based on UPLC-Q-TOF/MS and GC × GC-TOF/MS.

The expression levels of *GS* and *GOGAT* involved in N acquisition and assimilation increased by 42% and 78% under high-nitrogen relative to low-nitrogen conditions, respectively, in young shoots, whereas the level of *GDH* decreased by 39%. Furthermore, the expression levels of *GS* and *GOGAT* increased by 56% and 85% in roots, and *GDH* decreased by 19%. The expression levels of *PAL* related to secondary metabolism were downregulated by 51% and 4% in young shoots and roots, respectively. The expression levels of most genes involved in flavonoid metabolism (e.g., *DFR*, *ANR*, and *ANS*, among others) decreased with increasing N supply in young shoots. However, in roots, the expression levels of *DFR*, *ANR*, and *ANS* increased as the N supply increased. This result suggests that there are metabolic differences between shoots and roots (Figure 1).

### Correlation Analyses Between Metabolites and Key Genes

To investigate the correlations between metabolites and genes involved in the young shoots and roots of tea, we calculated the differences in metabolites and key genes in these tissues using Pearson correlation coefficients; then we created a correlation network using Cytoscape 2.82 (see text footnote 1). There were 406 correlations between genes and metabolites in young shoots (Supplementary Figure 2), of which 258 were negative, accounting for 63.5% of all correlations. Furthermore, we found that most genes involved in secondary metabolism correlated negatively with amino acids in young shoots (Supplementary Figure 2), which suggests that genes related to secondary metabolism could be inhibited as nitrogen metabolism increases under high nitrogen supply than under low nitrogen supply. However, there were 373 correlations between genes and metabolites in roots, of which 224 were positive, which accounts for 60.1% of all correlations. Most of the genes involved in secondary metabolism were positively correlated with amino acids in roots, indicating that roots and shoots have distinct temporal adaptation patterns in relation to different nitrogen supplies in tea (Supplementary Figure 2).

## Discussion

### Metabolite and Gene in Young Shoots Are Correlated With Carbon and Nitrogen Metabolism in Roots and Mature Leaves

**FIGURE 2:**
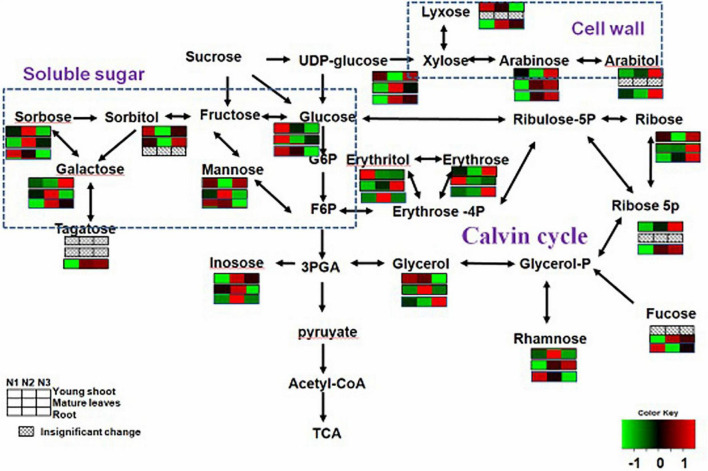
Changes in carbohydrates of young shoots, mature leaves, and roots under low (0.3 mmol/L, N1), intermediate (1.5 mmol/L, N2), and high levels of nitrogen (4.5 mmol/L, N3). The relative abundance of metabolites is illustrated on a red (high) to green (low) scale. The differences were considered to be significant when *p* < 0.05.

**FIGURE 3:**
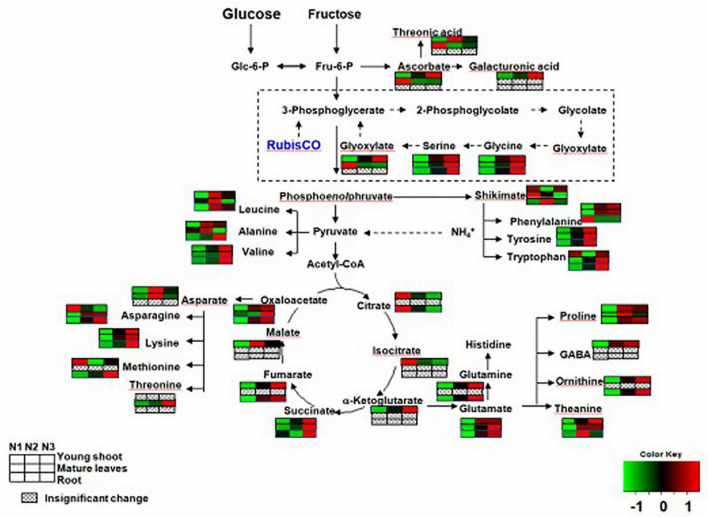
Changes in free amino acids and organic acids of young shoots, mature leaves, and roots under low (0.3 mmol/L, N1), intermediate (1.5 mmol/L, N2), and high levels of nitrogen (4.5 mmol/L, N3). The relative abundance of metabolites is illustrated on a red (high) to green (low) scale. The differences were considered to be significant when *p* < 0.05.

By investigating distinct parts of tea plants—i.e., the young tea shoots and mature leaves and the roots—our results suggested that the effects of N supply were inconsistent among those tissues. Carbon assimilation-related genes have been reported highly induced under conditions of low nitrogen supply (Schlüter et al., 2012). In our study, N-deficiency leads to an increase in several soluble sugars and sugar alcohols (e.g., glucose and sorbose) in young shoots. However, we noted that the contents of some sugars (e.g., mannose and glactose) increased in shoots but decreased in mature leaves and roots, which suggests that C is transported from the source (mature leaves and roots) to the sink (young shoots) (Figure 2). we also find that several carbohydrates involved in plant cell wall biosynthesis, such as galactose, arabinose, and xylose, were significantly increased in young shoots under high nitrogen supply versus under low nitrogen (Figure 2 and Supplementary Table 1). This result suggests that plant cell wall biosynthesis was modified (Burget et al., 2003; Linster and Clarke, 2008; Krapp and Daniel, 2011) under low nitrogen supply. In a previous study, a significant decrease was observed in the amount of a number of organic acids involved in the TCA cycle and carbon metabolism under conditions of low nitrogen supply (Ornston, 1971; Tschoep et al., 2009; Amiour et al., 2012). Furthermore, there is a remarkably positive correlation between the expressions of *GS* and *GOGAT* and the content of fumarate and succinate in young shoots and roots (Supplementary Figures 2A,B). Nitrogen assimilation and carbon metabolism are highly interconnected C skeletons and energy, supplied by photosynthesis, is used to produce amino acids (Krapp and Traong, 2006; Krapp and Daniel, 2011). In this study, organic acids (e.g., malate, fumarate, ketoglutarate, and succinate) involved in the tricarboxylic acid (TCA) cycle, which provides carbon skeleton for amino acid biosynthesis (Amiour et al., 2012), remarkably increased in the whole plant with increases in nitrogen supply (Figure 3 and Supplementary Table 1). The decreased C/N ratio in young shoots with increasing N supply shows that C is partitioned into compounds containing N (Ruan and Sattelmacher, 2007; Ruan et al., 2010). We found that a decrease in the organic acids involved in the TCA cycle was accompanied by increases in soluble sugars in young shoots under low nitrogen supply, simultaneously allowing amino acids related to nitrogen metabolism to decrease dramatically. Nitrogen and carbon transported from the source (mature leaves and roots) to the sink (young shoots) profoundly affect the accumulation of quality-related compounds in young tea shoots, such as amino acids.

Previous studies have shown that levels of amino acids and proteins in plant tissue significantly increase as nitrogen supply increases (Näsholm and Ericsson, 1990; Warren and Adams, 2000). In this study, the accumulation of free amino acids in all three organs (young shoots, mature leaves, and roots) depended largely on the N status, decreasing significantly in plants with low N supply (Table 1). Theanine in mature leaves and roots is the principal contributor to the increase in total amino acids in young shoots, in accordance with other free amino acids (glutamine, asparagine, glutamic acid, and alanine), acting as a dominant N transport from mature leaves and roots to young shoots during the sprouting period of the young shoots (Oh et al., 2008; Ruan et al., 2010). The biosynthesis of amino acids was affected by changing the N supply, and the synthesis of theanine was highly augmented when the external N supply increased (Supplementary Table 1). Ammonium is incorporated into glutamine and glutamate by glutamine synthetase and the glutamate synthase enzyme system (GS/GOGAT cycle) (Buchanan and Gruissem, 2000; Ferreira et al., 2016). Our results also show that the relative gene expressions (*GS* and *GOGAT*) with regard to the assimilation of nitrogen significantly increased in young shoots and roots with increasing nitrogen supply, suggesting that the GS/GOGAT cycle was activated. Furthermore, the evidence is increasing that GDH may reflect an additional or alternative route for the *GS/GOGAT* pathway for ammonia assimilation (Skopelitis, 2006; Krapp and Daniel, 2011). Notably, the expression of *GDH* was significantly inhibited under high nitrogen conditions in young shoots and roots, which is conflict with previous studies that show the GDH gene of tea plants was significantly upregulated by N supply, no matter supplied with NH_4_^+^ and NO_3_^–^ form (Huang et al., 2018; Yang et al., 2020). Conceding that three GDH genes (*Cs*GDH1-3) in tea plant has been reported previously and only one (TEA009809.1, *cs*GDH2) has been analyzed in this study, the roles of GDH1, GDH2 and GDH3 should be further analysed under different N supply (Tang et al., 2021). Furthermore, we found that the expression of *GS* and *GOGAT* had a significantly positive relationship with free amino acids (e.g., theanine, glutamine, and glutamate) in young shoots and roots (Figure 1 and Supplementary Figures 2A,B). The expression of *GDH* is negative for these amino acids, which signifies a good direct association between the gene involved in nitrogen absorption (*GS* and *GOGAT*) and these free amino acids in these tissues. Interestingly, we found that the tryptophan content was highest under low nitrogen supply in young shoots. Considering biosynthesis of IAA, a derivative of tryptophan, has been reported induced by low nitrogen supply in *Arabidopsis* (Ma et al., 2014), conditions of low nitrogen and high nitrogen both stimulated the biosynthesis of tryptophan in young shoots which may be how the tea plant adapts to abiotic stress.

### Change in Secondary Metabolites Under Different Nitrogen Supplies

**FIGURE 4:**
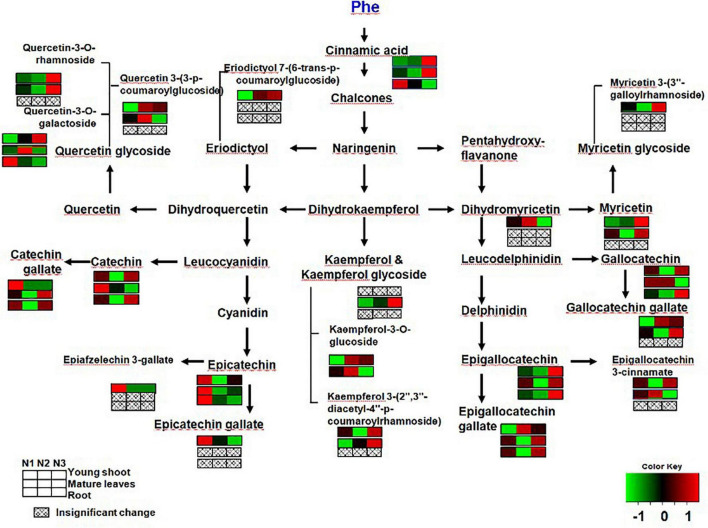
Changes in secondary metabolites of young shoots, mature leaves, and roots under low (0.3 mmol/L, N1), intermediate (1.5 mmol/L, N2), and high levels of nitrogen (4.5 mmol/L, N3). The relative abundance of metabolites is illustrated on a red (high) to green (low) scale. The differences were considered to be significant when *p* < 0.05.

Previous studies have found that gene expression in different branches of the flavonoid pathway is manipulated by nutrient depletion. For example, *CHS* (Bongue and Phillips, 1995) and *DFR* and *ANS* (Lillo et al., 2008) are strongly induced by nutrient deficiency, while PAL activity is downregulated and flavonoid accumulation is reduced under high N in young apple leaves (Strissel et al., 2005). In this study, the contents of CG, ECG, and EC significantly decreased more in young shoots under a high nitrogen supply than under low nitrogen supply (Figure 4). Ruan et al. (2010) found that the concentration of phenylalanine and PAL activity increased with increasing N supply. In our study, gene expression involved in flavonoid synthesis (e.g., *PAL* and *DFR*) was significantly upregulated under low nitrogen supply compared to high nitrogen in young shoots (Figure 1 and Supplementary Table 1), which suggests that the expression of genes involved in flavonoid synthesis is inhibited under high nitrogen supply. There might be a mechanistic link between genes involved in flavonoid synthesis and accumulation of polyphenols (Ruan et al., 2010) (Supplementary Figure 2). Furthermore, the flavonoid components generated from different metabolic branches were mediated by the nitrogen supply in shoots. For example, the content of ECG was negatively correlated with the content of quercetin-3-O-rhamnoside and quercetin-3-O-galactoside.

By analyzing the correlation between gene expression and metabolites, we found a significant negative relationship between gene expression (e.g., *PAL*, *CHI*, *CHS*, *CHS*, *DFR*, and *F3H*) involved in flavonoid synthesis and the content of free amino acids (e.g., phenylalanine, theanine, glutamine, and glutamic acid) in young shoots (Supplementary Figure 2 and Supplementary Table 1). This demonstrates increasing deviation of C flux to amino acids under conditions of abundant N supply in shoots (Ruan et al., 2010). Furthermore, the correlation network between flavonoid metabolites in young shoots was much closer than that in roots, which proves that flavonoid metabolism is expressed more actively in young shoots than in roots (Supplementary Figures 2, 3). Notably, high N supply significantly reduced the concentrations of most flavonoids in young shoots, while the total C concentration unchanged under high N supply. Moreover, some soluble sugars show lower levels under high N treatment while organic acids from TCA cycle increased. Those results indicate that the C flux has been optimized under N status changed.

The biosynthesis of phenolic compounds competes with protein synthesis for phenylalanine (Jones and Hartley, 1999). By analyzing the correlations among different metabolites in young shoots, we found that glucose and ECG were negatively correlated with free amino acids (e.g., phenylalanine, theanine, glutamine, and glutamic acid) (Supplementary Figure 3). This means that the balance shifted toward an increasing synthesis of amino acids associated with enhanced growth, while the investment of C in secondary metabolites was inhibited in shoots as nitrogen supply increased (Ruan et al., 2010).

## Conclusion

We analyzed the metabolite profile of young shoots, mature leaves, and roots grown under different nitrogen levels. We found that enhanced assimilation of N in tea roots and the coordinated regulation of carbon (sugars, organic acids, and flavonoids) and nitrogen (amino acids) in mature leaves can lead to a high accumulation of amino acids in young shoots. Furthermore, with increasing N supply, more C is partitioned into compounds containing N in mature leaves and roots, resulting in a decrease in flavonoids in young shoots.

## Data Availability Statement

The original contributions presented in the study are included in the article/Supplementary Material; further inquiries can be directed to the corresponding author.

## Author Contributions

JL, QZ, and ML gathered samples. QZ participated in the study design. JL, HF, QZ, and ML performed the data analysis. QZ and JL interpreted the results and drafted the manuscript. JR conceived of the study, provided funding, and gave guidance on experimental design. All authors read and approved the final manuscript.

## Conflict of Interest

The authors declare that the research was conducted in the absence of any commercial or financial relationships that could be construed as a potential conflict of interest.

## Publisher’s Note

All claims expressed in this article are solely those of the authors and do not necessarily represent those of their affiliated organizations, or those of the publisher, the editors and the reviewers. Any product that may be evaluated in this article, or claim that may be made by its manufacturer, is not guaranteed or endorsed by the publisher.

## Footnotes

1
http://www.cytoscape.org/
