# Supplementary material for: Accumulation of Amino Acids and Flavonoids in Young Tea Shoots Is Highly Correlated With Carbon and Nitrogen Metabolism in Roots and Mature Leaves
Source: Front Plant Sci. 2021 Nov 18;12:756433. doi: 10.3389/fpls.2021.756433 (PMC8636729; doi:10.3389/fpls.2021.756433)
Supplement: Supplementary file 2 [file Table_2.DOCX]

Table S2 Primer sequences for quantitative RT-PCR

| **Genes** | **Forward Primer (5’ - 3’)** | **Reverse Primer (5’- 3’)** | |
| --- | --- | --- | --- |
| 4CL | CGTGGTCCTCAAATTATG | | CATCATCGTCGTCTACAT |
| ANR | GCGAAGTTGATCCTCTCGTC | | AACCACATCGTCAAGTGAACA |
| ANS | TAATGGCAAGTACAAGAG | | CAATGGCTTCAAGATAATC |
| C4H | CAATGGCAATGACTTTAG | | CTCAGCAGTATCAATCTT |
| CHI | GTGATGGATGAAGTTGTG | | AAGAGAGAAAGCAGAGTC |
| CHS | TTACTAATAGCGAGCATAAGGT | | CTAGCATCAAGCGAAGGT |
| DFR | AGTTGTGTCGTTCTCATC | | GTATCAATGGCTCCTCTG |
| F3’5’H | AATCCTGGTGAAGAGAAG | | TCTATTATGCTTGATGATGTG |
| F3’H | ACCTTTCGACTTCACCCATCAAC | | TAACTGGACCATACGCAACCCTA |
| F3H | GCGACAGTATACCCCCTGAA | | AGTATGGCAAAGGCACATCC |
| FLS | GGAGAACAGCAAGGATATCG | | TCTCCTCCTGTGGGAGCTTA |
| LAR | GGGGCATCCTGTATCAAAGA | | CCGCATACCTTTCAGTCCAT |
| PAL | ATGACTTCTACAACAATGG | | GGAGTTCTGAGCAATAAG |
| GDH | GGCTCAACTAATGACCTGGAAG | | ACTCACTCTTACTTAACTCACTTGG |
| GOGAT | GAGGCGTGATGTATGTAACTGATA | | GGAACCTGCTAAACCACAATAAAC |
| GS | ACCAACTACAGTACCAAGTCCAT | | CCATAAGCAGCAATGTGTTCCT |
| GAPDH | TTGGCATCGTTGAGGGTCT | | CAGTGGGAACACGGAAAGC |
