# Supplementary material for: Accumulation of Amino Acids and Flavonoids in Young Tea Shoots Is Highly Correlated With Carbon and Nitrogen Metabolism in Roots and Mature Leaves
Source: Front Plant Sci. 2021 Nov 18;12:756433. doi: 10.3389/fpls.2021.756433 (PMC8636729; doi:10.3389/fpls.2021.756433)
Supplement: Supplementary file 3 [file Data_Sheet_1.docx]

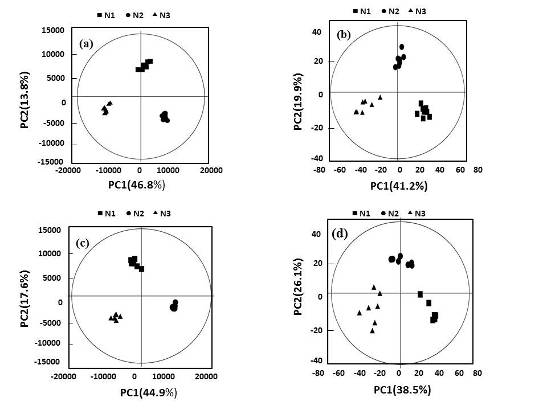

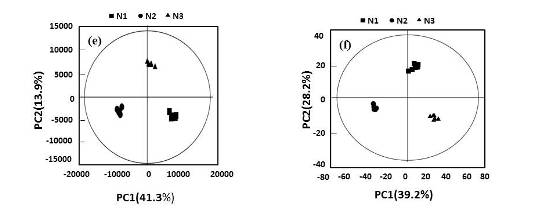


**Figure 1S.** PCA score plots derived from metabolites (a, b) in young shoots, metabolites (c, d) in mature leaves, and metabolites (c, d) in roots under low (0.3 mmol/L, N1), intermediate (1.5 mmol/L, N2), and high levels of nitrogen (4.5 mmol/L, N3). The nutrient solution is based on (a) UPLC-Q-TOF/MS and (b) GC×GCTOF/MS.

**
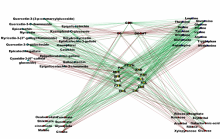

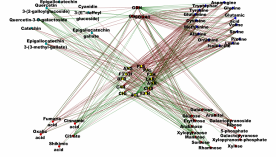
**

**a) Young shoots b) Roots**

**Figure 2S.** Map of significant gene-metabolite correlations. The ligature between metabolites and genes indicates their correlation. Positive correlations are shown in red, and negative correlations are shown in green.

**
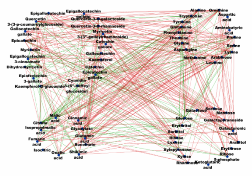

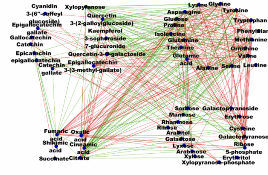
**

**a) Young shoot b) Root**

**Figure 3S.** Map of significant metabolite–metabolite correlations. The ligature between metabolites and gene indicates their correlation. Positive correlations are shown in red and negative correlations are shown in green.
